# Erythrocyte sedimentation rate, C-reactive protein, and interleukin-6 as inflammatory biomarkers in dogs naturally infected with *Ehrlichia canis*

**DOI:** 10.14202/vetworld.2021.2325-2331

**Published:** 2021-09-04

**Authors:** Thanaporn Asawapattanakul, Tanagorn Pintapagung, Supawadee Piratae, Siriluck Juntautsa, Pawarat Chancharoen

**Affiliations:** 1 Oxidative Stress Research Unit, Faculty of Veterinary Sciences, Mahasarakham University, Maha Sarakham 44000, Thailand; 2 Veterinary Clinic Research Unit, Faculty of Veterinary Sciences, Mahasarakham University, Maha Sarakham 44000, Thailand; 3 One Health Research Unit, Faculty of Veterinary Sciences, Mahasarakham University, Maha Sarakham 44000, Thailand; 4 Faculty of Veterinary Sciences, Mahasarakham University, Maha Sarakham 44000, Thailand.

**Keywords:** canine monocytotropic ehrlichiosis, C-reactive protein, erythrocyte sedimentation rate, interleukin-6

## Abstract

**Background and Aim:**

Canine monocytotropic ehrlichiosis (CME), a tick-borne disease, leads to a systemic inflammatory response syndrome; it is thus important to assess the intensity of inflammation in order to treat it appropriately. The current study was designed to evaluate hematological, biochemical, and inflammatory parameters in dogs naturally infected with *Ehrlichia canis* compared with those in healthy dogs. We also assessed the relationship among several inflammation-related parameters and considered these parameters for use as inflammatory biomarkers of CME.

**Materials and Methods:**

Twenty-eight dogs were divided into two groups based on the results of nested polymerase chain reaction for detecting *E. canis*, comprising a healthy group (n=11) and an infected group (n=17). A blood sample was collected from each dog to evaluate hematological, biochemical, and inflammatory parameters, with the obtained results being statistically compared between the groups. Moreover, the correlations of erythrocyte sedimentation rate (ESR), C-reactive protein (CRP), and interleukin-6 (IL-6) were investigated in the 28 dogs.

**Results:**

In the infected group, the mean levels of red blood cells, hemoglobin, and hematocrit were significantly lower than in the healthy group, while the mean lymphocyte and monocyte counts were higher. The mean levels of ESR and CRP were significantly higher (p<0.05) in the infected group, whereas no significant differences were found in IL-6 levels between the two groups. In the correlation analysis, ESR and CRP levels were highly correlated (p<0.01, r=0.531).

**Conclusion:**

Elevated ESR and CRP levels were found in dogs naturally infected with *E. canis*, which also presented mild to moderate inflammation in this study. Moreover, CRP was significantly correlated with ESR, so ESR and CRP may serve as inflammatory biomarkers for monitoring CME.

## Introduction

Canine monocytotropic ehrlichiosis (CME) is an important tick-borne infectious disease, which affects many dogs worldwide, including in Thailand [1]. CME is caused by Rickettsiales bacteria (*Ehrlichia canis*) and is characterized by various clinical signs depending on the stage of disease, such as fever, weight loss, anorexia, oculonasal discharge, hepatomegaly, splenomegaly, lymphadenopathy, bleeding disorder, epistaxis, ocular lesions, and neurological disorders [2,3]. Anemia and thrombocytopenia are also commonly diagnosed as hematological abnormalities in this disease [4]. The virulent stage of CME is associated with many complications, such as renal failure, acute pancreatitis, myocardial injury, immune-mediated hemolytic anemia, and bleeding disorder, leading to hypovolemic shock or disseminated intravascular coagulopathy [5-10]. These severe complications are associated with various inflammatory mediators such as cytokines, acute-phase protein, nitric oxide, and oxidative stress, resulting in a systemic inflammatory response syndrome. Therefore, to treat this disease in an appropriate manner, it is important to assess the intensity of inflammation for diagnosis and to monitor the severity of the disease [11,12].

Erythrocyte sedimentation rate (ESR) test is an inexpensive evaluation performed by measuring the distance that red blood cells (RBC) descend in a test tube (Westergren tube) within 1 h. A high ESR is found in inflammatory conditions due to a large increase in fibrinogen levels; it is usually used as a generic index of illness and for tracking inflammation in humans. ESR can be used to identify various diseases and conditions, such as acute tissue injury, infection, malignancy, rheumatoid arthritis, lymphoma, coronary disease, stroke, and heart failure, as well as for postoperative monitoring [13-17]. ESR has rarely been analyzed in dogs, but it has been found to be useful in cases of canine rheumatoid arthritis, osteoarthritis, and babesiosis [18-20]. Moreover, acute-phase proteins, especially C-reactive protein (CRP), produced and released by the liver, are also used for the clinical diagnosis and monitoring of inflammatory diseases. CRP levels are known to increase significantly during acute inflammatory processes and host responses to infection [21]. In dogs with acute CME, CRP concentrations were reported to increase dramatically between days 4 and 16, peaking at 1-6 weeks after inoculation with *E. canis* in dogs; similarly, most dogs with chronic CME from natural infection presented an increase of CRP levels. Thus, CRP concentration may increase in CME-affected dogs with both acute and chronic clinical signs [11,22]. Interleukin-6 (IL-6) is a cytokine or inflammatory mediator that can be used as a biomarker of various diseases. Measurement of its level can be useful for diagnosis and prognostication in both infectious and non-infectious inflammatory diseases, in humans (COVID-19 and total knee replacement surgery), and animals (canine end-stage renal disease and babesiosis) [23-26]. Although IL-6 has been shown to have many positive effects on various inflammatory diseases in animals, studies on this issue in CME have been limited. It was previously reported that CRP can be useful for tracking inflammation as a parameter for predicting severity in various canine inflammatory disorders including CME [11,12,22]. ESR and IL-6 (IL-6 stimulating the hepatic synthesis of CRP) have not often been considered for use as clinical inflammatory biomarkers of animals, and there have been few publications on these parameters in CME-affected dogs.

This study was established to compare hematological and serum biochemistry indices, as well as the intensity of inflammation, using ESR, CRP, and IL-6 levels, between dogs naturally infected with *E. canis* and healthy dogs. We also assessed the relationship among several inflammatory parameters and considered the value of using these parameters as inflammatory biomarkers of CME.

## Materials and Methods

### Ethical approval and Informed consent

The research protocol and animal experiments were granted ethical approval by the Institutional Animal Care and Use Committee of Mahasarakham University, Thailand (Ethics approval number: IACUC-MSU-10/2021), and written informed consent of owners was obtained before starting the research.

### Study period and location

The study was conducted from October to May 2021 in the district of Mueang, Maha Sarakham province, Thailand.

### Patients

The inclusion criteria for animal selection in this study were as follows: canine patients (client-owned dogs) of either sex and any breed, weighing >5 kg, and aged between 1 and 5 years. However, we excluded any patients if they presented infection with *Babesia* spp. or *Anaplasma* spp., or exhibited inflammatory conditions such as liver disease, renal disease, dermatitis, wound, or skin neoplasia [27].

All dogs that fulfilled the specific criteria were evaluated for their hematological and biochemical profiles, and were analyzed for *E. canis* infection using clinical signs, thin blood smear, rapid enzyme-linked immunosorbent assay (ELISA, SNAP^®^ 4Dx^®^ Plus; IDEXX Laboratories, Westbrook, ME, USA), and nested polymerase chain reaction (PCR) using *E. canis*-specific primers (*16s rRNA* gene). The dogs were then divided into two groups: a healthy group and an infected group. The criteria for inclusion in each group in this study were as follows:

The healthy group: The dogs showed no notable clinicopathological signs of *E. canis* infection, normal hematological and biochemical profiles, and negative results for *E. canis* by thin blood smear, SNAP 4DX® Plus test (IDEXX Laboratories, USA), and PCR assay (n=11).

The infected group: The dogs with and without clinical signs showed positive results for *E. canis* by thin blood smear, SNAP 4DX® Plus test (IDEXX Laboratories), and PCR assays (n=17).

### Blood collection and hematological and plasma biochemistry profiles

Blood samples were collected from the cephalic vein and then divided into ethylenediaminetetraacetic acid (EDTA) tubes and blood serum separator tubes. Each EDTA whole-blood sample was separated for PCR (frozen at -20°C), and the remaining blood was prepared within 2 h for CBC and ESR tests, while plasma serum samples from blood serum separator tubes were used for biochemical analysis (measurements of CRP and IL-6 levels). Hematological and plasma biochemical profiles were analyzed using an IDEXX ProCyte Dx^®^ automated hematology analyzer (IDEXX Laboratories) and the TC200 Fully Automatic Chemistry Analyzer (TECOM TC220; TECOM, Jiangxi, China).

### Molecular detection of E. canis

For molecular detection in this study, nested PCR was used following a previously reported protocol [4]. DNA was extracted from 200 mL of each EDTA whole-blood sample with the GF-1 Blood DNA Extraction Kit (Vivantis, Malaysia). The concentration of extracted DNA was measured using a NanoDrop 1000 spectrophotometer (Thermo Scientific, Wilmington, DE, USA) at a wavelength of 260 nm. A set of oligonucleotide primers for nested PCR was used to amplify the *16s rRNA* gene sequences of *E. canis*, as previously described by Bulla *et al*. [28]. The nucleotide sequences of the universal primers of rickettsia for the initial reaction were as follows: ECC 5’- AGAACGAACGCTGGCGGCAAGCC-3’ and ECB 5’-CGTATTACCGCGGCTGCTGGCA-3’. For the later step of PCR, the following *E. canis*-specific primers were used: CANIS 5’-CAATTATTTATAGCCTCTGGCTATAGGA-3’ and HE3 5’-TATAGGTACCGTCATTATCTTCCCTAT-3’ [4,28]. In the first step of PCR, the PCR mixture with a volume of 25 mL consisted of 50 ng of the purified DNA, 10pmol of each primer, 200 mM each dNTP, 1.5 mM MgCl_2_, and 1 U of Taq DNA polymerase (Vivantis). PCR amplification for the identification of *E. canis* was performed using a Biometra GmbH thermocycler (Germany) and consisted of the following schedule: 2 min at 95°C; 35 cycles of 45 s of denaturation at 95°C, 45 s of annealing at 60°C, and 90 s of extension at 72°C; and then final extension at 72°C for 5 min. The results of ethidium bromide-stained agarose gel electrophoresis of PCR products were identified under ultraviolet light [4].

### ESR

Three milliliter EDTA-anticoagulated whole-blood samples obtained by venipuncture were prepared for the measurement of ESR. The Westergren method used in this study is a specific and standard test for determining ESR based on the method of the International Council for Standardization in Hematology. For this method, a commercial ESR pipette was placed vertically at room temperature (18-25°C) away from direct sunlight, draught, and vibration, and the distance (millimeters in 1 h) that the RBCs descended from the start of the test were measured. ESR test was conducted within 2 h of sample collection [29].

### CRP

The CRP concentrations in the serum of dogs were determined using a commercial dry chemistry slide (IDEXX Catalyst^®^ CRP Test). Each sample was analyzed by IDEXX Catalyst One^®^ chemistry analyzer (IDEXX, Westbrook, ME, USA) [30,31].

### Serum IL-6

The quantitative measurement of serum cytokine (canine IL-6) was performed using Abcam’s IL-6 Canine ELISA Kit (ab193686; Abcam, USA), which is an ELISA. A 96-well plate was prepared for the cytokine assay, in accordance with the manufacturer’s instructions. Each canine IL-6 standard or serum (100 mL) was pipetted into a well, in which IL-6 samples were bound to the primary capture antibody coated on the 96-well plate. Then, the biotinylated anti-canine IL-6 antibody (primary detector antibody, 100 mL) was added, producing an antibody–antigen–antibody “sandwich.” HRP-conjugated streptavidin (100 mL) was subsequently pipetted into the wells, followed by 100 mL of TMB One-Step Substrate Reagent. The plate was incubated for 30 min at room temperature in the dark before adding 50 mL of stop solution in the final step. The absorbance was then read at 450 nm using a microplate reader (Infinite^®^ M Nano, Tecan, Switzerland). The concentrations of serum IL-6 were calculated using a standard curve.

### Statistical analysis

Data analysis was performed using IBM SPSS Statistics Version 22 (IBM, Chicago, IL, USA). The obtained data, including hematological and biochemical profiles, ESR levels, and the concentrations of CRP and IL-6 were calculated and presented as mean ± standard error for each variable and group of dogs. Independent t-tests were performed to compare and evaluate the statistical significance of the differences in means between the infected group (*E. canis*-infected dogs) and the healthy group. Correlations among inflammatory markers were calculated using Pearson’s correlation<analysis. Differences were statistically significant at p<0.05.

## Results

Samples of dogs were divided into two groups, comprising a healthy group (n=11) and a infected group (n=17). The means of hematological and biochemical values obtained from the two groups were compared for significant differences (at p<0.05). The important hematological findings were normocytic normochromic anemia, thrombocytopenia, lymphocytosis, and monocytosis, as shown in Table-1.

**Table 1:** Hematological and plasma biochemistry indices of healthy and infected groups.

Parameters	Unit	Healthy group	Infected group	p-value
	
		Mean±SE	Mean±SE	
WBC	×10^3^/µL	12.34±0.94	14.17±0.79	0.153
RBC	×10^6^/µL	7.44±0.29	5.42±0.28	0.000*
Platelet	×10^3^/µL	195.09±20.93	47.24±13.15	0.000*
Hemoglobin	g/dL	16.60±0.66	11.46±0.61	0.000*
Hematocrit	%	46.74±2.00	33.09±1.67	0.000*
MCV	fL	62.75±0.83	61.24±0.75	0.196
MCH	pg	22.31±0.24	21.16±0.30	0.010*
MCHC	g/dL	35.55±0.23	34.55±0.24	0.008*
Neutrophil	×10^3^/µL	7.26±0.69	6.73±0.44	0.502
Lymphocyte	×10^3^/µL	2.92±0.41	4.79±0.46	0.009*
Monocyte	×10^3^/µL	0.69±0.08	1.55±0.19	0.002*
Eosinophil	×10^3^/µL	1.41±0.20	1.16±0.19	0.388
Basophil	×10^3^/µL	0.05±0.01	0.06±0.01	0.775
ALT/SGPT	U/L	33.00±8.73	34.81±3.39	0.828
creatinine	mg/dL	1.45±0.06	1.25±0.06	0.944

* p<0.05, SE=Standard error, WBC=White blood cells, RBC=Red blood cells, MCV=Mean corpuscular volume, MCHC=Mean corpuscular hemoglobin concentration, ALT=Alanine aminotransferase, SGPT=Serum glutamic pyruvic transaminase

**Figure-1:**
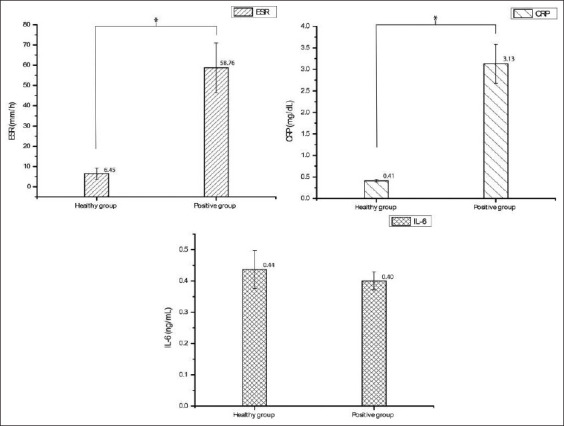
Comparison of inflammatory biomarkers comprising ESR, serum CRP, and IL-6 in the healthy group and infected group (*Ehrlichia*-infected dogs).

In the present study, the mean levels of ESR, CRP, and IL-6 were compared between the healthy and infected groups. No statistically significant difference in IL-6 level was identified (p>0.05); by contrast, the mean levels of ESR and CRP differed significantly (p<0.001) between the two groups. The mean ESR and CRP levels in the infected group were approximately 8 times higher than those in the healthy group (58.76 vs. 6.45 mm/h, and 3.12 vs. 0.4 mg/dL, respectively), as shown in Figure-1. In the descriptive statistics, ESR and CRP levels were categorized into three subgroupsaccording to levels of intensity, while IL-6 levels were divided into two subgroups. The results of the ESR subgroupsin healthy and infected groups revealed that the subgroup with ESR of less than 10 mm/h accounted for 90.91% in the healthy group and 3% in the infected group, while the subgroups with ESR of 10-60 mm/h and more than 60 mm/h constituted almost half of the total in the infected group (both 41.18%). Regarding the CRP level, all dogs in the healthy group had a CRP concentration in serum of <1 mg/dLwhile in the infected group, the subgroup with a CRP level of more than 3 mg/dL constituted the highest subgroup (52.94%). However, in healthy and infected groups, serum IL-6 concentrations of <0.4 ng/mL were the most common, shown by 63.64% of subjects in the healthy group and 64.71% in the infected group. The data are presented as the percentage of subjects in each subgroup in Table-2.

**Table 2:** Comparison of inflammatory markers divided subgroups in the healthy group and infected group (*Ehrlichia*infected dogs).

ESR level (mm/h)	Healthy group	Infected group	CRP level (mg/dL)	Healthy group	Infected group	IL-6 level (ng/mL)	Healthy group	Infected group
					
	n (%)	n (%)		n (%)	n (%)		n (%)	n (%)
<10	10 (90.91)	3 (17.65)	<1	11 (100.00)	2 (11.76)	<0.4	7 (63.64)	11 (64.71)
10-60	1 (9.09)	7 (41.18)	1.0-3.0	0 (0.00)	6 (35.29)	>0.4	4 (36.36)	6 (35.29)
>60	0 (0.00)	7 (41.18)	>3.0	0 (0.00)	9 (52.94)			
Average (mean±SE)	6.45±2.79	58.76±12.30		0.41±0.03	3.13±0.45		0.44±0.06	0.40±0.03
p-value	0.001			0.000			0.552	
95% CI	0.23-12.68	32.70-84.83		0.34-0.48	2.17-4.09		0.30-0.57	0.34-0.46

SE=Standard error, CI=Confidence interval

Table-3 shows the results of correlation analysis among the clinical inflammatory markers ESR, CRP, and IL-6. A significant correlation (p=0.004, r=0.531) was identified between ESR and CRP, while there were no significant correlations of IL-6 with ESR and IL-6 with CRP.

**Table 3:** Correlations among clinical inflammatory marker levels in healthy and CME-infected dogs.

Measure	Statistical values (r)	p-value
ESR-CRP	0.531	0.004*
ESR-IL6	−0.233	0.232
CRP-IL6	0.015	0.941

* p<0.05, CME=Canine monocytotropic ehrlichiosis, ESR=Erythrocyte sedimentation rate, CRP=C-reactive protein, IL6=Interleukin 6

## Discussion

This study compared hematological and biochemical profiles between healthy dogs and naturally CME-infected dogs. The mean levels of RBC, hemoglobin, and hematocrit in the infected dogs were significantly lower than those in the healthy dogs. According to the results on these parameters obtained in this study, although the majority of naturally infected dogs presented mild anemia (normocytic normochromic anemia), some dogs presented normal values of RBC parameters (data not shown), as previously reported by Thongsahuan *et al*. [32]. Consistent with the previous reports, the normocytic normochromic anemia identified in this study may have been caused by immune-mediated disorders and bone marrow suppression [33,34]. Thrombocytopenia was found in dogs of the CME group in this study, while healthy dogs presented normal platelet levels, in agreement with a previous study [4]. Furthermore, the mechanisms of thrombocytopenia in ehrlichiosis in this study may have involved decreased production, increased consumption and destruction, splenic sequestration, bleeding disorders, and inflammation. It has been reported that decreased platelet production is frequently associated with ehrlichiosis and rickettsial diseases by bone marrow suppression [35]. Immune-mediated thrombocytopenia, also known as autoimmune thrombocytopenia or idiopathic thrombocytopenia, is related to the increased consumption and destruction of platelets through the production of autoantibodies against platelet autoantigens and decreases in platelet half-life and the number of platelets. Moreover, immune-mediated vasculitis, coagulopathy, and platelet dysfunction were suggestive of underlying thrombocytopenia associated with natural ehrlichiosis, resulting from vessel wall injury [8,36]. Splenomegaly and splenic sequestration have been reported as pathological findings of CME because the spleen is the main source of *Ehrlichia*-infected macrophages, leading to platelet destruction through associated immune and inflammatory responses [37,38]. Here, it was found that there were significant differences in monocytosis and lymphocytosis between the infected group and the healthy group, in line with a previous study [39].

In recent years, many publications have suggested that IL-6 is useful as an inflammatory marker for various diseases and likely to increase the acute-phase response during inflammation, especially in tick-borne diseases [40,41]. In our study, there was no significant difference (p>0.05) in serum IL-6 concentration between the healthy and CME-infected groups. Moreover, the previous studies reported no increases in IL-6 level in CME dogs, in line with our study. This may have been caused by the strain-specific cytokine response or duration of infection [37,42,43]. Alternatively, other studies on CME reported increases in the levels of other cytokines such as IL-1b, IL-8, IL-10, and tumor necrosis factor-alpha (TNF-a) [37,42]. Increased CRP levels in the infected group in our study were likely to have been caused by IL-1b or TNF-a stimulated in the acute-phase response, in accordance with the previous studies [42,44]. Our results showed that the mean levels of CRP and ESR in the infected group were significantly higher than those in the healthy group (p<0.05). The average CRP concentration in the *Ehrlichia*-infected group in this study was approximately 8 times higher than that of healthy dogs, and was similar to the results of previous studies [11]. Moreover, the *Ehrlichia*-infected group in this study demonstrated a marked increase in ESR, as in the previous reports on infections with the blood-borne pathogens *Ehrlichia* spp. and *Babesia* spp. [20,45]. CRP is produced by hepatocytes in the acute-phase response to inflammatory stimuli due to many factors including ehrlichiosis. It was reported that CRP levels rapidly increased from 6 h after an inflammatory trigger and peaked at 48 h, after which they gradually dropped to the normal range [46]. The slow replication of *E. canis* might be the cause of the delayed injury or inflammation found between 3 and 4 weeks after infection, and CRP concentrations may therefore increase in both acute and chronic CME [11,22]. As mentioned above, CRP measurements can be of value clinically and are sensitive for detecting CME or for monitoring the severity of inflammation, especially in the early stage of disease. Alternatively, an increase of ESR levels depends on the augmentation of fibrinogen levels in the blood. ESR levels began to increase within 24 h, peaked in 2 weeks, and gradually returned to the baseline thereafter [47]. Although ESR is not as sensitive as CRP due to it having a delayed response to inflammation, it is a cheap and reliable option for use as a test for screening CME.

In this study, ESR level was positively correlated with CRP level, which is in line with previous results from some inflammatory diseases such as rheumatic disease [48]. Although many studies have reported that IL-6 level was correlated with ESR or CRP level in various inflammatory conditions, in the present study on CME no correlations of IL-6 with ESR and IL-6 with CRP were identified [49,50]. Considering each variable in the current study, the ESR and CRP levels in the healthy dogs were almost in the normal ranges (<10 mm/h and 1 mg/dL, respectively), while those in the CME group were significantly higher. The ESR values were used to divide the CME-affected dogs into three subgroups as follows: <10 mm/h, 10-60 mm/h, and >60 mm/h. The majority of CME-affected dogs were classified into the 10-60 mm/h subgroup (41.18%) and >60 mm/h subgroup (41.18%). Similarly, the concentration of CRP was revealed to be high (>3 mg/dL; moderate inflammation) in most CME-affected dogs (52.94%), while the intermediate concentration subgroup (1-3 mg/dL; mild inflammation) was the second largest (35.29%) [46]. According to the levels of CRP and ESR in this study, the infected dogs presented CME of mild to moderate severity.

## Conclusion

This study presents a comparative study of the hematological profiles in two groups of dogs with and without *E. canis* infection. Normocytic normochromic anemia, thrombocytopenia, lymphocytosis, and monocytosis were hematological abnormalities found only in the dogs infected with *E. canis*. This study also revealed a significant correlation between CRP and ESR. Significant increases in CRP and ESR were found in the dogs naturally infected with CME. These findings on CRP and ESR indicate their potential value as inflammatory biomarkers for monitoring CME.

## Authors’ Contributions

TA: Designed and performed the experiment, collected and analyzed the data, and wrote the manuscript. TP: Designed and performed the experiment, checked the data analysis, and revised the manuscript. SP: Performed the experiment, revised the method, and critically revised the manuscript. SJ: Performed the experiment and critically revised the manuscript. PC: Performed the experiment and critically revised the manuscript. All authors read and approved the final manuscript.
